# The Challenges of Spinal Surgery Requiring Ventral Decubitus in the Critical Trauma Patient on Extracorporeal Membrane Oxygenation: A Case Report

**DOI:** 10.7759/cureus.45904

**Published:** 2023-09-25

**Authors:** Patrícia Martins Lima, Sérgio G Pinto, José Dias

**Affiliations:** 1 Anesthesiology, Centro Hospitalar Universitário São João, Porto, PRT

**Keywords:** trauma anesthesia, acute respiratory distress syndrome [ards], traumatic spinal fracture, prone position surgery, vv ecmo

## Abstract

Extracorporeal membrane oxygenation (ECMO) provides a bypass of the lungs, ensuring blood oxygenation and carbon dioxide removal in cases of respiratory failure. The nature of the device itself creates many perioperative challenges, including fluid management and the management of anticoagulation.

Surgery via the posterior approach for an unstable spinal fracture requiring the ventral decubitus position comes with its own set of difficulties, among which are the need for stability and craniocaudal alignment when rotating the patient, the risk of increased abdominal pressure, and the damage to vulnerable soft tissues like the eyes, nose, and others.

The combination of these two situations creates a synergistic effect, which adds to the difficulty of the management of the situation and requires a personalized, multidisciplinary approach.

We present a case of a critical trauma patient who was on venovenous ECMO as a consequence of refractory respiratory hypoxemia with an unstable mid-thoracic spinal fracture requiring surgical intervention via the posterior approach (demanding intra-operative ventral decubitus).

## Introduction

Extracorporeal membrane oxygenation (ECMO) is a form of prolonged cardiopulmonary bypass that involves the diversion of venous blood through a circuit comprising a blood pump and a gas-exchange device (membrane oxygenator) before returning it to the arterial or venous circulation. ECMO provides both oxygenation and carbon dioxide removal, thereby bypassing the pulmonary circulation and allowing lung rest.

Venovenous (VV) ECMO is used for isolated respiratory failure. Blood is drained from the venous system and returned to the venous system after oxygenation. This modality provides respiratory support but does not offer hemodynamic support, while venoarterial (VA) ECMO is used for combined respiratory and cardiac failure, providing both oxygenation and circulatory support [1].

Indications for ECMO include severe refractory hypoxemia, hypercapnic respiratory failure, or cardiogenic shock unresponsive to conventional treatments [2].

Surgery that requires posterior access to the patient requires positioning in the ventral decubitus. This position has many known risks and associated complications, as described by Kwee et al. in their 2015 review [3]. Especially relevant to the case at hand are the potential increase in intra-abdominal pressure and potential vascular compromise, interference with cardiovascular function, misalignment of the spine leading to neurologic injury, and pressure-related injuries to soft tissues like the eyes and nose.

Surgical interventions for patients on ECMO present a unique constellation of difficulties [2]. The delicate balance between hemorrhage and thrombosis and the management of anticoagulation remain controversial [4]. The optimization of respiratory dynamics and potential blood gas derangements despite the ECMO are important risks. Moreover, physical factors, namely the positioning of the patient, surgical draping, and the surgical approach itself, can inadvertently disrupt the ECMO cannulae or compromise flow dynamics, thereby affecting the efficacy of oxygenation and perfusion.

## Case presentation

**Figure 1:**
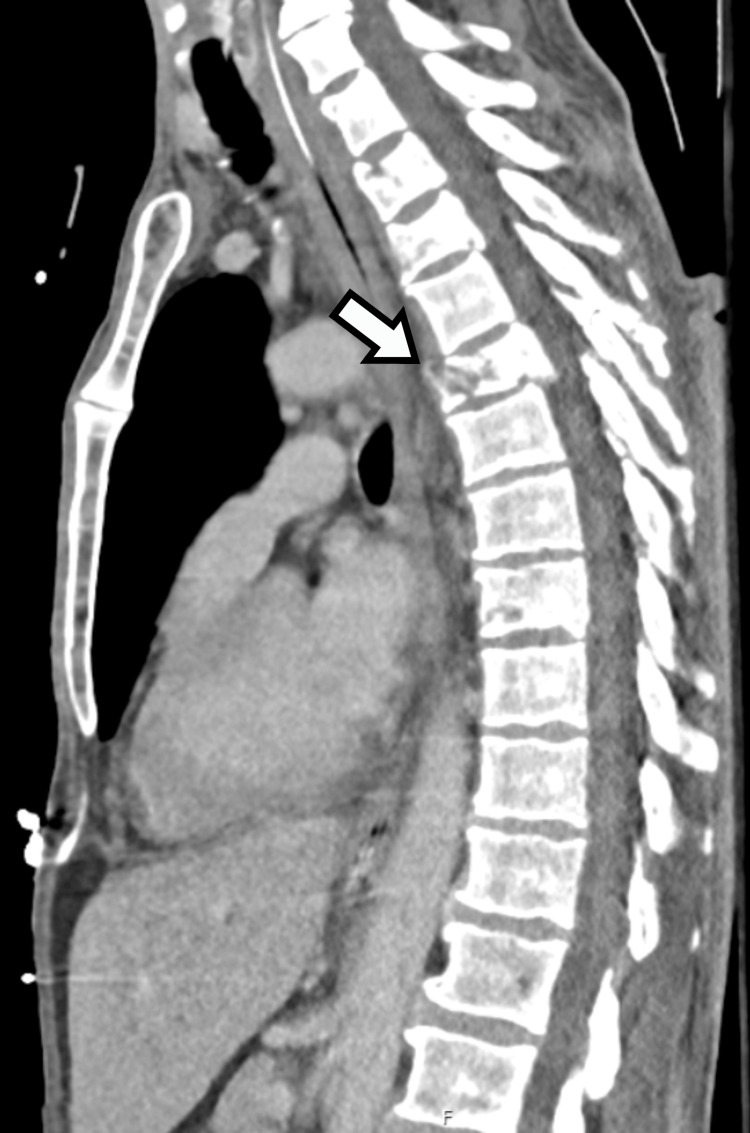
Spinal CT scan in the ER Arrow: T5 with a complete burst fracture. CT: Computerized tomography; ER: Emergency room

The patient described in this case is a 59-year-old male with a history of chronic alcoholism and no other relevant medical history. He was admitted to the emergency room (ER) of a local secondary hospital after falling from a height of 7 meters onto the pavement. The diagnostic investigation in the ER revealed multiple injuries, namely vertebral trauma with an unstable, complete burst fracture of T5 with posterior bulging into the medullary canal (Figure 1) and stable fractures of T1-3; thoracic trauma with pneumothorax, five right costal fractures and three left costal fractures, and multiple pulmonary contusions; abdominal trauma with splenic and hepatic laceration; and pelvic trauma with multiple pelvic fractures.

The patient was admitted to the intensive care unit (ICU) for continuation of care, under mechanical ventilation, and with an airway secured by an endotracheal tube.

**Figure 2:**
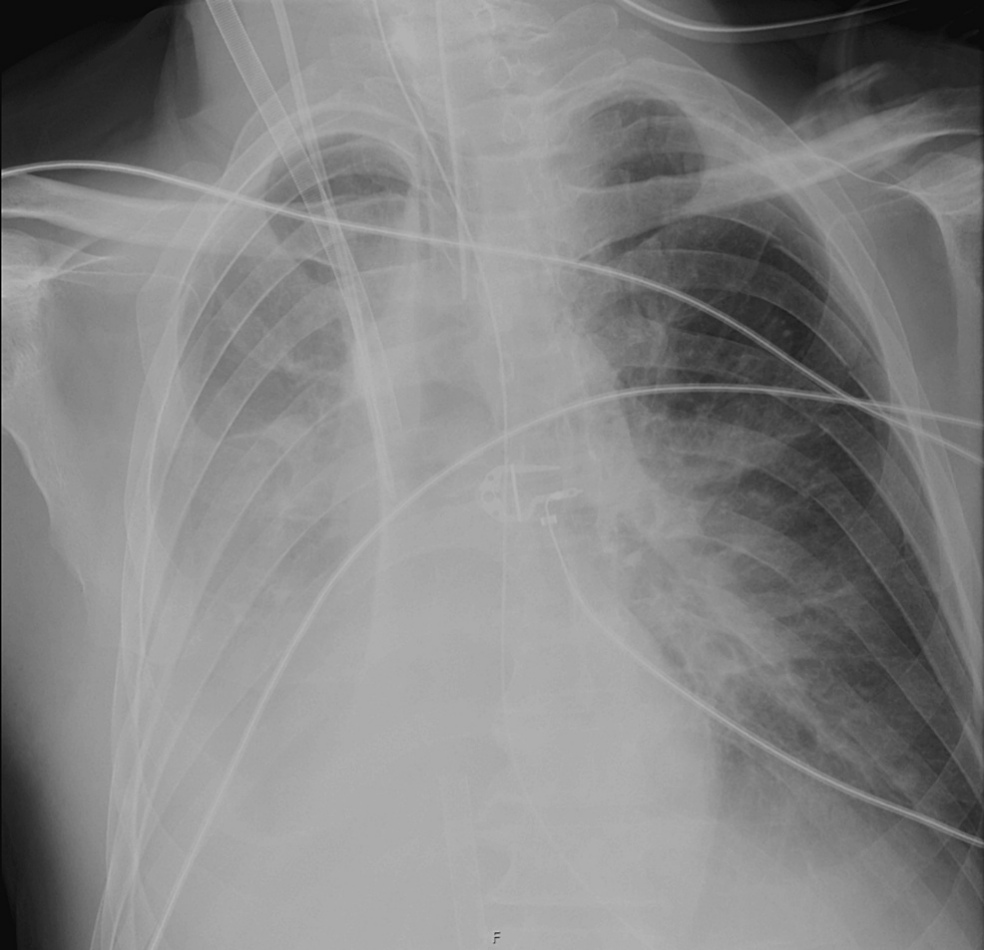
The patient's X-ray in the ICU ICU: Intensive care unit

During the five days spent in this ICU, the patient developed acute respiratory distress syndrome (ARDS) and multiple, repetitive atelectasis superimposed on a pulmonary infection centered on the areas of pulmonary contusion (Figure 2). This led to the development of hypoxemic respiratory failure, that was highly refractory to conventional intervention. Attempting to rotate the patient to ventral decubitus to improve respiratory dynamics was not possible, given the presence of the unstable vertebral fracture. In light of this unfavorable clinical evolution and the lack of alternatives, the decision was made to submit the patient to ECMO rescue after an evaluation from general surgery, noting that the abdominal trauma did not contraindicate the anticoagulation necessary for ECMO. However, the local secondary hospital lacked ECMO capabilities.

The ECMO team of a regional tertiary hospital was contacted and traveled to the local secondary hospital to place the patient on a VV femoro-jugular (VV-FJ) ECMO using the Cardiohelp system (Maquet, Rastatt, Germany). The outflow cannula was inserted in the right femoral vein, and the inflow cannula was inserted in the right jugular vein. The patient was transported, accompanied by the ECMO team, to the ICU of the aforementioned tertiary hospital. Computerized tomography (CT) imaging studies confirmed the position of the cannulae, with the femoral cannula terminating at the confluence of the hepatic veins and the inferior vena cava and the jugular cannula terminating at the confluence of the brachiocephalic and the superior vena cava. The patient was evaluated by general surgery, who determined that the abdominal injuries did not require surgical intervention and did not contra-indicate surgery in ventral decubitus, and by orthopedics, who determined that the vertebral fractures required surgical fixation as soon as the patient’s status permitted.

While in the ICU, the patient was anticoagulated using a non-fractioned heparin (NFH) infusion, monitored with daily activated prothrombin time (aPTT) assays, and under sedoanalgesia with propofol, dexmedetomidine, fentanyl, and rocuronium infusions, with a target of a Richmond Agitation and Sedation Scale of -5.

The patient responded poorly to antibiotic therapy, and his respiratory status worsened, making ECMO withdrawal unfeasible. After a multidisciplinary discussion, including the ICU physicians, the clinical perfusion team, the anesthesiology team, the orthopedics team, and the general surgery team, the decision was made to proceed with surgical fixation of the vertebral fractures while the patient was still on ECMO. A contingency plan was established for the surgery, outlining the roles of each team member intraoperatively as well as the steps to be taken in case of intraoperative ECMO dysfunction.

The surgical plan was an internal fixation of the T1-3 and T5 vertebrae via a posterior approach.

The patients’ NFH infusion was suspended for eight hours before surgery. The values of aPTT were 45.3 s before suspension and 36 s in the immediate pre-operative period. Baseline hemoglobin was 8.1 g/dL, and the patient had required the transfusion of a total of four units of red blood cell concentrate (RBC) up to this point.

Upon arrival at the operating room (OR), the patient was placed under total intravenous general anesthesia (TIVA) using propofol, fentanyl, and rocuronium infusions. The endotracheal tube was replaced with an armored endotracheal tube under direct visualization by video laryngoscopy (C-MAC) and using a Frova endotracheal tube introducer (Cook Medical, Bloomington, IN, USA). The patient was monitored according to the American Society of Anesthesiologists Standards for Basic Anesthetic Monitoring, as well as invasive blood pressure monitoring via an arterial catheter in the right brachial artery; neuromuscular blockade monitoring with a kinemyiography train-of-four (TOF) device; anesthetic depth monitoring with a processed electroencephalogram monitor (Bispectral Index (BIS) (Medtronic, Minneapolis, MN, USA); brain oxygenation monitoring with the INVOS (Medtronic, Minneapolis, MN, USA) near-infrared spectroscopy (NIRS) system; central temperature monitoring with an esophageal probe; and urinary output monitoring via urinary catheter.

Venous access to the patient was assured with a triple-lumen central venous catheter in the right jugular vein, as well as three additional peripheral 16-G venous catheters.

The patient remained under a protective ventilation strategy using the pressure-controlled volume-guaranteed modality, with a tidal volume of 6 mL/kg (with a total tidal volume of 420 mL for a predicted body weight of 70,42 kg) and a positive end-expiratory pressure of 8 mmHg.

**Figure 3:**
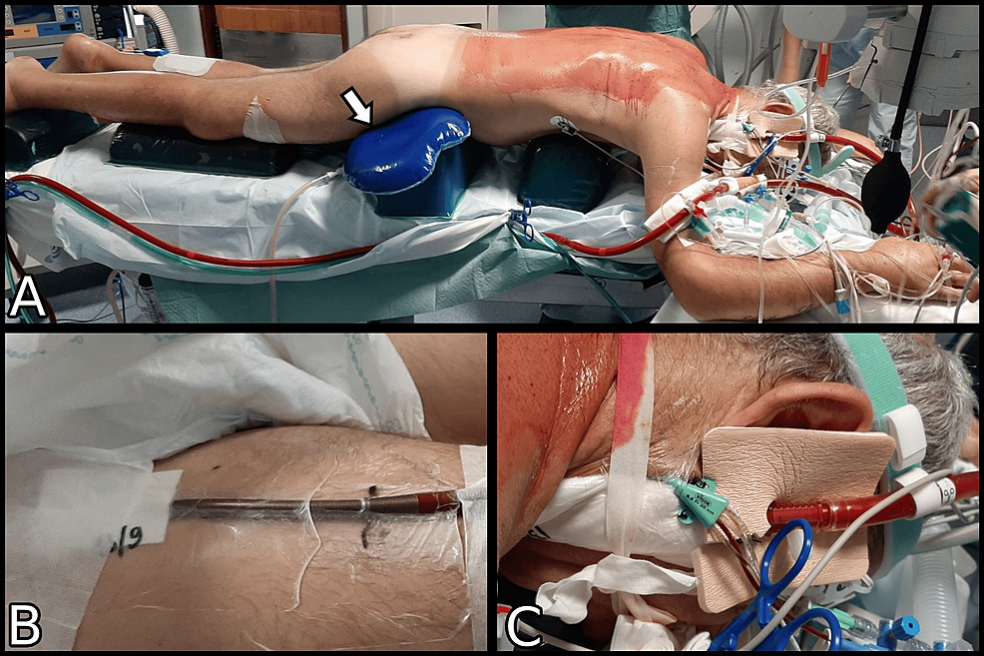
Patient positioning before surgical incision A: Global view of the patient in ventral decubitus position before surgical incision. Arrow: Additional gel cushion in the femoral cannula insertion site. B: Femoral cannula insertion site, viewed in dorsal decubitus. C: Jugular cannula insertion site, viewed in ventral decubitus.

The orthopedics, anesthesiology, and clinical perfusion teams were present in the OR throughout the surgery. The patient was rotated en bloc to the ventral decubitus position. The anesthesiologist remained at the head of the patient and was responsible for stabilizing the neck and giving movement commands. The clinical perfusion specialist supervised the rotation and paid special attention to ensuring the integrity of the ECMO cannulae. The remaining team members performed the rotation technique while ensuring alignment of the central bony axis of the patient. Gel cushions were utilized to position the patient while ensuring that the abdomen, axillae, eyes, and nose remained free of pressure and that sensitive pressure points, such as the bony prominences of the hip, knees, ankles, and elbows, were adequately protected and cushioned. Additional cushioning was added to protect the insertion sites of the ECMO cannulae, as per the direction of the clinical perfusion team. In Figure 3, we can see the patient in the final ventral decubitus position immediately before the start of surgery.

The VV-FJ ECMO was configured with the following settings: blood flow rate 4.12 Lpm; 3200 rpm; sweep rate 2.5 Lpm. The oxygenator was replaced immediately before transport to the OR.

During the surgery, the clinical perfusion specialist alerted the team to an episode of ECMO dysfunction, identified as a sudden decrease in flow rate and revolutions per minute. There was no impact on ΔP or SvO2. In accordance with our contingency plan, this triggered a temporary pause in the surgical procedure to investigate and resolve the issue. The situation was resolved with the repositioning of the gel cushions under the femoral cannula and abdomen, with normal flow being resumed rapidly and with no further dysfunction, namely, without an impact on the patient’s blood pressure or brain oxygenation. This event occurred a total of three times during the surgery. 

At the end of the surgery, the patient was rotated back to dorsal decubitus with the same precautions as before. There were no surgical complications.

The surgery lasted 211 minutes. Ventilation was satisfactory, and normocapnia was assured, with a peak inspiratory pressure below 40 cmH2O throughout. The patient required vasopressor support with a noradrenaline infusion at a dose of 0.05 ug/kg/min, maintaining hemodynamic stability with an average mean arterial pressure of 77 mmHg and a mean heart rate of 93 bpm. The patient suffered a total quantifiable fluid loss of 875 mL, divided between urinary losses of 475 mL and hemorrhagic losses of 400 mL, and was administered 2000 mL NaCl 0.9% and 500 mL of RBC for a total positive fluid balance of 1625 mL. Worsening anemia, identified in serial arterial blood gas measurements, required the infusion of two RBCs. Brain oxygenation remained symmetrical and within 20% of the baseline values defined at the start of the surgery. TOF measurements were consistent at 0 responses. Normothermia was assured, with a mean temperature of 36.1 ºC. The mean blood glucose was 104 mg/dL. 

**Figure 4:**
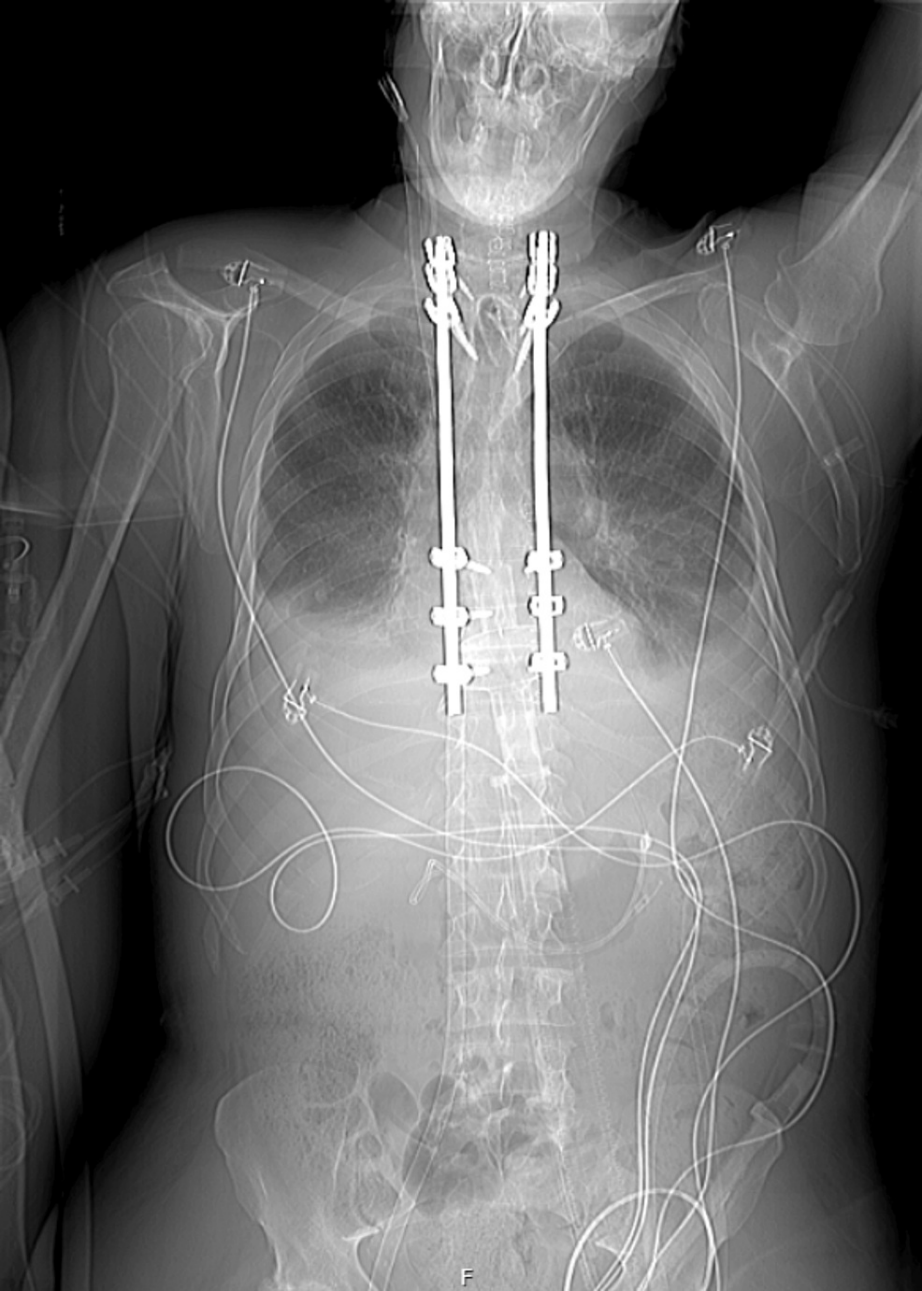
The patient's post-operative X-ray

During the surgery, 1000 mg of propofol, 50 mg of rocuronium, and 525 ug of fentanyl were administered. There were no surgical complications. A post-operative X-ray shows successful fixation of the fractured vertebrae, as seen in Figure 4.

The patient returned to the ICU under mechanical ventilation. Given the anticipation of a long period of mechanical ventilation and the convenience of having already suspended NFH, the patient was submitted to a tracheostomy in the immediate post-operative period, replacing the armored endotracheal tube with a 9-mm non-fenestrated tracheal cannula.

NFH was restarted 24 hours postoperatively. The value of aPTT was 38.9 s before the infusion was restarted and returned to therapeutic levels 24 hours later. There were no thromboembolic complications.

ECMO withdrawal was successfully achieved 17 days after the surgery, the cannulae were removed 18 days after the surgery, and ventilatory withdrawal and tracheal decannulation were achieved 36 days after the surgery. Hospital discharge was 50 days after the surgery, with no hemorrhagic complications and no further respiratory complications throughout his inpatient stay, with the patient being transferred to a secondary care facility under the care of orthopedics and physical medicine to begin his rehabilitation.

Six months after discharge, the patient was satisfied with the outcome of his treatment and, while limited, was making significant progress in the recovery of his autonomy with the assistance of a comprehensive rehabilitation program. The patient suffered some neurologic injuries as a result of his spinal trauma, with permanent urologic dysfunction requiring chronic urinary catheterization.

## Discussion

This paper describes one of the few cases of a surgical procedure requiring ventral decubitus performed on a patient who is on ECMO. It serves to highlight the difficulties and potential pitfalls and proposes potential strategies to overcome these issues, ensuring ideal outcomes for the patient.

The case at hand combines the difficulties of performing surgery on a critical patient who was on ECMO with an unstable spinal fracture at risk of neurologic damage in the ventral decubitus position. The risk of interference with the ECMO circuitry is exacerbated in this position, be it because of the risk of increased intra-abdominal pressure or because of the risk of hemorrhage and hypovolemia. Manipulating the patient, both to rotate him in the ventral decubitus position and to operate, carries a risk of decannulation that cannot be underestimated, and the unstable nature of the fracture being operated on in this case meant that any and all mobilizations had to be carried out with maximum care and stability to avoid neurologic damage [5].

These difficulties make undertaking a surgical procedure that requires the ventral decubitus position in a patient on ECMO an undesirable first choice. For this reason, an attempt was made to withdraw the patient from ECMO before the surgical intervention. This attempt was, however, unsuccessful. The presence of an unstable vertebral fracture at a mid-thoracic level posed a very significant risk of neurologic injury and required absolute immobility, which in practice made it impossible to optimize the patients’ ventilation and respiratory status, worsening the atelectasis, limiting respiratory physical therapy, and ultimately creating a positive feedback loop that worsened the patient’s condition. When it became clear that it would not be possible to resolve the patients’ underlying issues while these limitations were present, the decision was made to proceed with the surgery despite these anticipated difficulties.

While our hospital is a national reference center for ECMO, this was our first surgical procedure demanding ventral decubitus in a patient on ECMO. The lynchpin of our management of this case was the establishment of a multidisciplinary contingency plan on how to deal with adverse events involving the ECMO during surgery, with clear roles and responsibilities for each member of the team, as follows: upon identifying an issue with the ECMO, the clinical perfusion specialist present in the OR would immediately notify the remaining members of the team, and surgery would (if possible) temporarily cease while the issue was investigated. If it were possible to resolve the issue with simple measures, such as repositioning the gel cushions, no further intervention would be necessary, and surgery would resume. However, should there be a need for further investigation, it could become necessary to temporarily close the surgical wound and rotate the patient back to the dorsal decubitus to expose the cannulae and restore flow. If necessary, the clinical perfusion team had equipment and professionals ready to re-cannulate the patient in the OR.

This strategy proved successful in identifying and resolving the issues that were encountered intraoperatively. 

The events of ECMO dysfunction that occurred intraoperatively manifested as a sudden reduction in flow rate with a negative impact on revolutions per minute. Analogously to the functioning of the heart, this translated into a reduction of pre-load in the ECMO device, pointing to a dysfunction of the femoral outflow cannula as the most likely cause. Ventral decubitus is associated with an increase in abdominal pressure, and surgical manipulation can exacerbate this [3], which can lead to compression of the inferior vena cava and reduce the available blood flow through the femoral cannula. Line kinking and cannula insertion site obstructions were also a possibility, albeit less likely, given the armored nature of the cannulae used. Hypovolemia could also have a similar presentation, but it would be unlikely to resolve with positional measures. Prompt identification of these events and a rapid response from the team were paramount in guaranteeing that they had no impact on patient hemodynamics.

ECMO requires an adequate circulating volume to function, making fluid management and hemorrhagic control a priority [1]. In our case, the surgical hemorrhagic risk was high [6], and the patient presented with baseline anemia, exacerbating the risks. To guarantee adequate venous access for massive fluid and RBC resuscitation, the patient had a triple-lumen central venous catheter and three 16-G peripheral venous catheters. Given the patient’s underlying status and dysfunction, we aimed for a hemoglobin target of 8 g/dL [4,7], which required the intraoperative transfusion of two RBCs. Crystalloid fluids were used, with 2 L of NaCl 0.9% being infused during the surgery, achieving the goal of a positive intraoperative fluid balance.

Anticoagulation in ECMO is an area of great interest and development. While the majority of societal guidelines recommend that all patients on ECMO be on anticoagulants [4], the trend seems to be shifting towards less anticoagulation for patients on VV-ECMO, with a 2021 review by Olson et al. reporting (within its limitations as outlined in the paper) no differences in the incidence of thromboembolic events regardless of the use of anticoagulation [8]. NFH is the most commonly used anticoagulant in this context, and it is frequently monitored with the use of aPTT assays, as was the case in our patient. As mentioned above, the surgical hemorrhagic risk was high, and our patient had baseline anemia, in addition to the hemorrhagic risk inherent to being on ECMO. These factors, associated with the lesser thromboembolic risk associated with VV-ECMO, led us to suspend anticoagulation eight hours before surgery and to restart it 24 hours later.

The role of antifibrinolytic agents, such as aminocaproic or tranexamic acid, in patients on ECMO is the subject of research and analysis. A large 2005 review found that aminocaproic acid significantly reduced surgical site bleeding in pediatric ECMO patients [9]. In their 2021 paper, Coleman et al. describe a clinically significant reduction in bleeding, RBC transfusion requirements, and other hematological interventions with the use of aminocaproic in a pediatric ECMO cohort [10]. The use of antifibrinolytics, however, appears to have an association with increased formation of circuit thrombi [11], and there is a distinct lack of literature describing the use of antifibrinolytic agents in adult populations on ECMO. We opted not to use either of these agents on this patient. However, this is a question that, in the authors’ opinion, warrants further research and investigation.

Autologous blood transfusion (ABT) is the process of reinfusing the patient’s own blood, either collected and stored preoperatively or salvaged intraoperatively. It’s an important strategy for hemorrhagic mitigation, with increasing usage in ORs throughout the globe. By utilizing ABT, risks associated with allogeneic blood, such as transfusion-transmitted infections, hemolytic reactions, and transfusion-related acute lung injury (TRALI), are markedly reduced [12]. Moreover, there are important ancillary benefits, both in reducing the strain on heterologous blood supplies and in terms of financial costs. While it was not possible to use ABT in this case due to material limitations, the authors believe that it is an important component in perioperative blood management strategies.

Normothermia, as defined by a core body temperature within the bounds of 36.5°C to 37.5°C, plays an important role in perioperative hemorrhagic control. The coagulation cascade has several temperature-sensitive steps, and even mild hypothermic shifts can cause notable declines in the activity of clotting factors such as II, VII, IX, and X [13]. Hypothermia has an important negative effect on platelet function as well, as demonstrated by Wallner et al. in their 2022 study [14]. Ensuring normothermia through available means, namely, by limiting patient exposure and using convective air-warming systems and fluid-warming devices, plays an important role in minimizing hemorrhagic loss.

In addition to all the strategies discussed above, a meticulous approach by the surgeons themselves focused on minimizing surgical hemorrhagic loss is paramount to any perioperative blood management strategy. 

We decided to monitor brain oxygenation using a NIRS device, the INVOS. Evidence suggests that NIRS is a valuable aid in assessing regional blood flow in patients on ECMO, and given the concerns about potential ECMO dysfunction, it provided an additional source of information regarding the integrity of the system [15]. Ventral decubitus can have an impact on cerebral oximetry by itself [16], with Closhen et al. describing a positive effect of around 5% in a cohort of patients undergoing elective orthopedic surgery [17]. However, there are important confounding factors that must be taken into account, among which are the physical interference of gel cushions and other positioning devices with the INVOS sensors themselves [18].

Intraoperative transesophageal echocardiography (TEE) could have been a valuable addition to this patient’s intraoperative strategy by offering real-time imaging of cardiac structures and function, shedding light on potential changes in contractility or wall motion abnormalities indicative of myocardial ischemia as well as potential valvular pathologies or dysfunctions. TEE can also provide crucial insights into the positioning and functionality of the ECMO cannulae, ensuring any malposition or migration is promptly detected. Additionally, TEE is invaluable in assessing volume status and guiding fluid management [19].

However, TEE in a patient positioned in ventral decubitus is challenging [20]. Insertion of the probe in the ventral decubitus is difficult, and the process of rotating a patient with an inserted TEE probe can dislodge the probe. Once in the ventral position, the change in orientation might complicate the visualization of specific cardiac areas. The large size of the TEE probe can increase intra-abdominal pressure, acting synergically with the ventral decubitus position in this regard, potentially leading to difficulties in ventilation or compression of the inferior vena cava, exacerbating ECMO dysfunction events like the ones we encountered. Moreover, the unusual nature of conducting TEE in a ventral decubitus position cannot be overstated, and a trained TEE operator might grapple with the distinct challenges posed by this position.

Given these considerations, we opted to forego the use of TEE in this case. While TEE remains a potent tool, its application in certain situations, particularly in the ventral decubitus position, calls for deliberate and informed evaluation.

This paper, being a single case report, has a series of inherent limitations, namely, difficulty in generalizing our findings to the general population of patients on ECMO. The authors are of the opinion that further research is paramount in identifying the ideal strategies for these patients.

## Conclusions

This case reports on our approach and strategy to handle a rare confluence of difficult situations: a critically ill trauma patient with a multifactorial hypoxemic respiratory insufficiency under VV-FJ ECMO with an unstable thoracic vertebral fracture at risk of permanent neurologic injury requiring surgery in the ventral decubitus position. Anticipation is fundamental in atypical cases, and the perioperative, multidisciplinary establishment of a step-by-step contingency plan with the entire team’s responsibilities outlined allowed for rapid identification and resolution of intraoperative ECMO dysfunction.

Management of anticoagulation is challenging in these cases, as the inherent thromboembolic and hemorrhagic risks of ECMO are amplified by the risks associated with surgery, especially bleeding and hypovolemia. We describe a successful strategy of perioperative NFH suspension with aPTT monitoring, in line with current recommendations for VV-ECMO.
